# Post-translational modifications and protein quality control of mitochondrial channels and transporters

**DOI:** 10.3389/fcell.2023.1196466

**Published:** 2023-08-03

**Authors:** Ashlesha Kadam, Pooja Jadiya, Dhanendra Tomar

**Affiliations:** ^1^ Department of Internal Medicine, Section of Cardiovascular Medicine, Section of Molecular Medicine, Wake Forest University School of Medicine, Winston-Salem, NC, United States; ^2^ Department of Internal Medicine, Section of Gerontology and Geriatric Medicine, Section of Molecular Medicine, Wake Forest University School of Medicine, Winston-Salem, NC, United States

**Keywords:** mitochondrial transporters, mitochondrial channels, posttranslational modifications, MPQC, MCU, VDAC, MPTP, SLCs

## Abstract

Mitochondria play a critical role in energy metabolism and signal transduction, which is tightly regulated by proteins, metabolites, and ion fluxes. Metabolites and ion homeostasis are mainly mediated by channels and transporters present on mitochondrial membranes. Mitochondria comprise two distinct compartments, the outer mitochondrial membrane (OMM) and the inner mitochondrial membrane (IMM), which have differing permeabilities to ions and metabolites. The OMM is semipermeable due to the presence of non-selective molecular pores, while the IMM is highly selective and impermeable due to the presence of specialized channels and transporters which regulate ion and metabolite fluxes. These channels and transporters are modulated by various post-translational modifications (PTMs), including phosphorylation, oxidative modifications, ions, and metabolites binding, glycosylation, acetylation, and others. Additionally, the mitochondrial protein quality control (MPQC) system plays a crucial role in ensuring efficient molecular flux through the mitochondrial membranes by selectively removing mistargeted or defective proteins. Inefficient functioning of the transporters and channels in mitochondria can disrupt cellular homeostasis, leading to the onset of various pathological conditions. In this review, we provide a comprehensive overview of the current understanding of mitochondrial channels and transporters in terms of their functions, PTMs, and quality control mechanisms.

## 1 Introduction

Mitochondria are the cellular power station and signaling hub, consisting of a double membrane system that forms the outer mitochondrial membrane (OMM) and inner mitochondrial membrane (IMM), creating the intermembrane space (IMS) and mitochondrial matrix (MM). The OMM is porous, allowing free passage of molecules less than 10,000 Da, with no membrane potential across it. On the other hand, the IMM acts as a highly discerning barricade against small molecules and ions, permitting regulated passage exclusively through specific membrane transport/channel proteins (Gray, 2001; Kuhlbrandt, 2015). The IMM is divided into two distinct regions: the inner boundary membrane (IBM), which connects with the outer mitochondrial membrane (OMM), and the cristae membrane (CM), which forms inward-folded structures within the MM (Hoshino et al.). IBM and CM are functionally distinguished and have different protein compositions. The CM is enriched with respiratory chain complexes, iron-sulfur cluster assembly proteins, and selective channels/transporters while the IBM contains the protein translocation and membrane fusion machinery (Vogel et al., 2006; Suppanz et al., 2009). Cristae junctions are tube-like membrane invaginations of ∼25 nm diameter that separate IBM and CM (Rabl et al., 2009; Davies et al., 2012; Busch, 2020). The cristae occupy most of the inner membrane surface, indicating their crucial role in cellular physiology. Mitochondria are essential for various cellular processes such as oxidative phosphorylation, calcium (Ca^2+^) and redox regulation, mitochondrial protein folding, mitophagy, metabolic pathways such as tricarboxylic acid-, β-oxidation- and urea cycle TCA cycle, and controlling signaling events from cell survival to cell death (Manoli et al., 2007; Murphy et al., 2016). To carry out these high-energy-requiring cellular processes, the crosstalk between mitochondria and the cytoplasm occurs uni- or bi-directionally through various channels and transporters present on the mitochondrial membranes, which function as gatekeepers, restricting access to the organelle from the cytoplasm. This selectivity of both the OMM and IMM helps to maintain MM’s volume by regulating influx and efflux mechanisms for ions and metabolites, primarily K^+^ and Ca^2+^. Additionally, these fluxes help to balance the levels of other important ions such as Na^+^, Cl^−,^ and H^+^. Disruptions in ion homeostasis in the cytosol and MM can lead to detrimental consequences, ultimately causing cell death (Javadov et al., 2018).

Mitochondrial (∼99%) proteins are mostly encoded by the nucleus and equipped with specific targeting sequences that guide them from the cytosol to the mitochondrial surface receptors, ensuring correct protein localization and assisting in protein quality control. The translocases not only transport newly synthesized proteins to their correct mitochondrial destinations but also establish a complex dynamic network (Dolezal et al., 2006; Neupert and Herrmann, 2007). The efficiency of protein import into mitochondria is a significant indicator of the healthy energetic state of mitochondria. Dysfunction in mitochondrial metabolism decreases inner membrane potential (ΔΨm) and impairs protein import (Nunnari and Suomalainen, 2012; Frazier et al., 2019). The mitochondrial transport machinery plays a vital role in protein biogenesis, and its quality control is essential for understanding mitochondrial stress response-related diseases. The mitochondrial proteome quality is necessary for cellular survival and is maintained by various interlinked cellular processes (Calvo et al., 2016; Gómez-Serrano et al., 2018). The mitochondrial protein quality control system ensures proper protein production, maintenance, functional switching, and degradation (Anand et al., 2013). Various interlinked cellular processes, from the molecular to the organellar level, participate to maintain mitochondrial proteome/homeostasis based on cellular need and this is achieved by the mitochondrial protein quality control (MPQC) system (Jadiya and Tomar, 2020). The cytoplasmic protein quality control machinery helps eliminate misfolded proteins in the OMM, while each interior compartment of mitochondria (IMS, MM) possesses its own specialized machinery, including mitochondrial chaperones, proteases, and disaggregases (Baker and Haynes, 2011; Song et al., 2021). As mitochondria play a crucial role in cellular events, including essential pathways of amino acid biosynthesis, fatty acid oxidation, and signal transduction, stress response apoptotic pathways, maintaining a healthy mitochondrial environment is crucial for cell function (Schon and Manfredi, 2003). Therefore, uninterrupted checks and repair of mitochondria are necessary to keep a healthy mitochondrial pool in cells.

**FIGURE 1:**
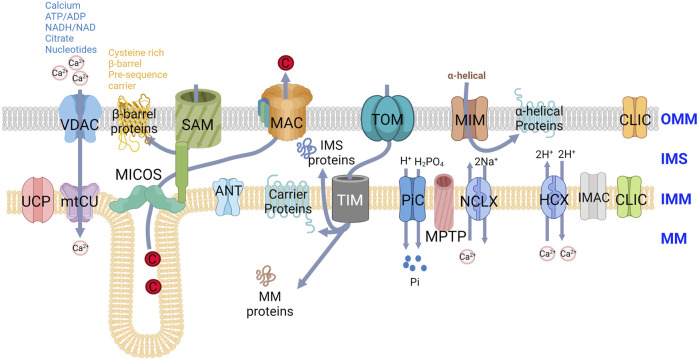
Channels and transporters present at the OMM and IMM. The transporters and channel proteins present on OMM and IMM are depicted with the exchange ion/molecule machineries. VDAC-Voltage-dependent anion channel; SAM- Sorting and Assembly Machinery; MAC-Mitochondrial Apoptosis-induced Channel; TOM-Translocase of the OMM; MIM-Mitochondrial import; CLIC-Mitochondrial Chloride (Cl^−^) channel; UCP- Uncoupling proteins; mtCU- Mitochondrial calcium uniporter channel; MICOS- Mitochondrial contact site and cristae organizing system; ANT-ADP, ATP translocase; TIM- Translocase of the Inner Membrane; PiC- Mitochondrial phosphate carrier; MPTP- MPermeability Transition Pore; NCLX- Na^+^/Ca^2+^/Li^+^ exchanger; HCX- H^+^/Ca^2+^ exchanger; IMAC-Mitochondrial inner membrane anion channel.

**FIGURE 2:**
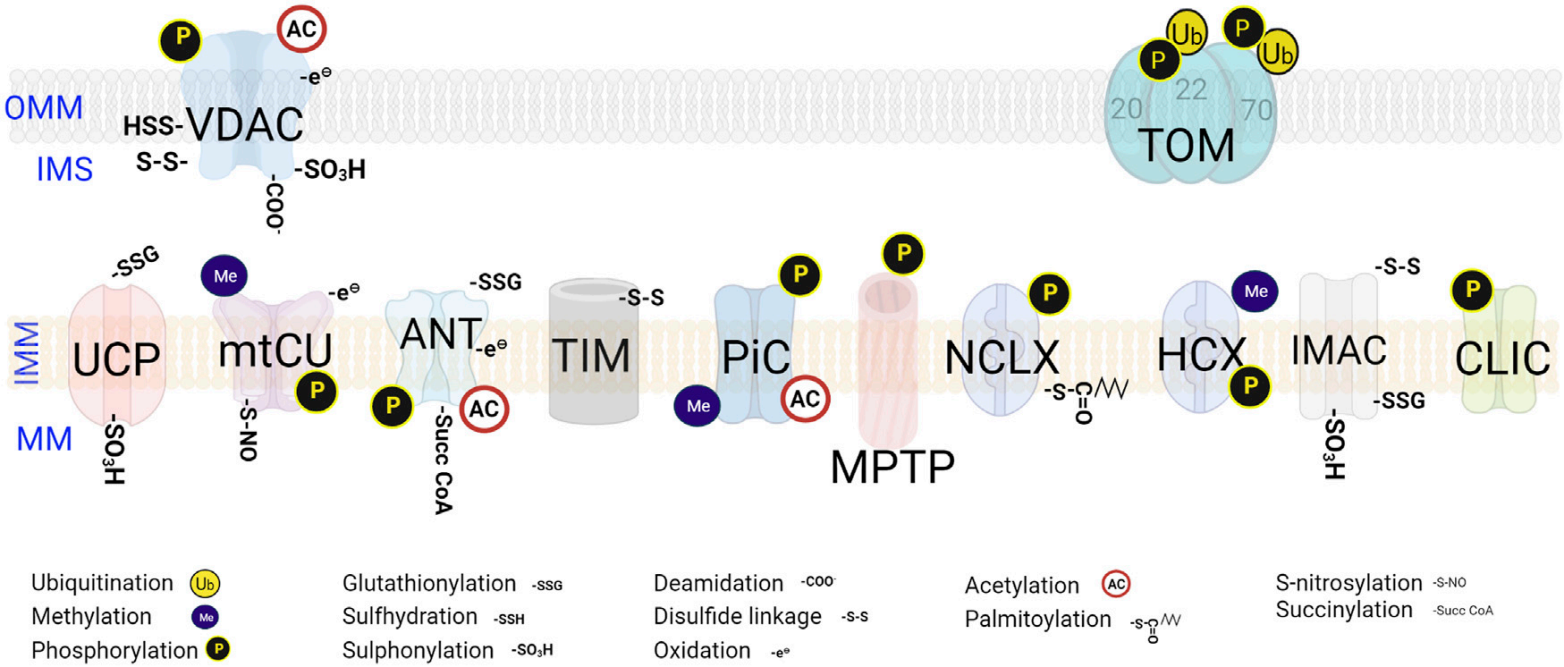
Post-translational modifications on mitochondrial channels and transporters. The PTMs occur at specific sites on the transporters. The various regulatory PTMs exhibited by both OMM and IMM transporters and channels are denoted. VDAC-Voltage-dependent anion channel; TOM-Translocase of the OMM; UCP- Uncoupling proteins; mtCU- Mitochondrial calcium uniporter channel; ANT-ADP, ATP translocase; TIM- Translocase of the Inner Membrane; PiC- Mitochondrial phosphate carrier; PTP- Permeability Transition Pore; NCLX- Na^+^/Ca^2+^/Li^+^ exchanger; HCX- H^+^/Ca^2+^ exchanger; IMAC-Mitochondrial inner membrane anion channel; CLIC-Mitochondrial Chloride (Cl^−^) channel.

Post-translational modifications (PTMs) are crucial for maintaining the proteome and mitochondrial homeostasis. Throughout the protein molecular transition process, which includes transcription, post-transcription, translation, and post-translation, mitochondrial channels and transporters are tightly regulated by PTMs. These modifications, such as acetylation, phosphorylation, ubiquitination, nitrosylation, and oxidation, alter the chemical properties of amino acid residues, thereby influencing protein distribution, stability, and function (Doyle and Mamula, 2001; Beltrao et al., 2013). Although the influence of PTMs on misfolded proteins is extensively studied, our understanding of the underlying molecular pathways governing these PTMs and their significance in mitochondrial proteins, specifically channels and transporters, is still incomplete. Moreover, the molecular identity of numerous mitochondrial channels and transporters has been recently discovered or remains unknown, which has limited exploration of their PTMs. In this review, we aim to present an up-to-date overview of various mitochondrial channels and transporters, along with their associated PTMs and MPQC mechanisms (Figures 1, 2), shedding light on their roles in both normal physiological processes and pathological conditions.

## 2 Mitochondrial transporters/channels

Mitochondrial transporters and channels play a critical role in regulating the exchange of metabolites between the MM and the cytoplasm while maintaining the electrochemical proton gradient required for oxidative phosphorylation (OXPHOS). These channels are located in both the OMM and IMM and the exchange of metabolites/ion fluxes is maintained by various chemical signals and mechanical stimuli, allowing metabolites compartmentalization and regulation of cell volume and ΔΨm (O'Rourke, 2007; Szabo and Zoratti, 2014). Understanding the molecular processes of mitochondrial channels and transporters is critical to comprehend their biological significance.

### 2.1 OMM channels/transporters, their control mechanisms, and PTMs

The OMM contains around 200 proteins that act as the first line of defense against the influx of metabolites into the mitochondria (Pfanner et al., 2019). The main transporters and channels present in the OMM, along with their PTMs and quality control mechanisms, are discussed in detail here.

#### 2.1.1 Translocation of proteins through OMM

##### 2.1.1.1 Translocase of the OMM (TOM) complex

The TOM complex serves as the main access point for proteins into the mitochondria. High-resolution cryo-electron microscopy studies show that it consists of three receptors, Tom20, Tom22, and Tom70, the channel forming unit, Tom40, and three small proteins, namely, Tom5, Tom6, and Tom7. Tom20 and Tom70, the peripheral TOM receptors, bind to incoming proteins and recognize pre-sequence or mitochondrial localization sequences. The main subunit of the TOM complex is Tom40, the β-barrel protein which can form a protein-conducting channel. Tom22 is the docking site for Tom20 and Tom70, enabling protein precursor transfer from the receptor toward the translocation channel (Wang et al., 2020; Guan et al., 2021).

**FIGURE 3:**
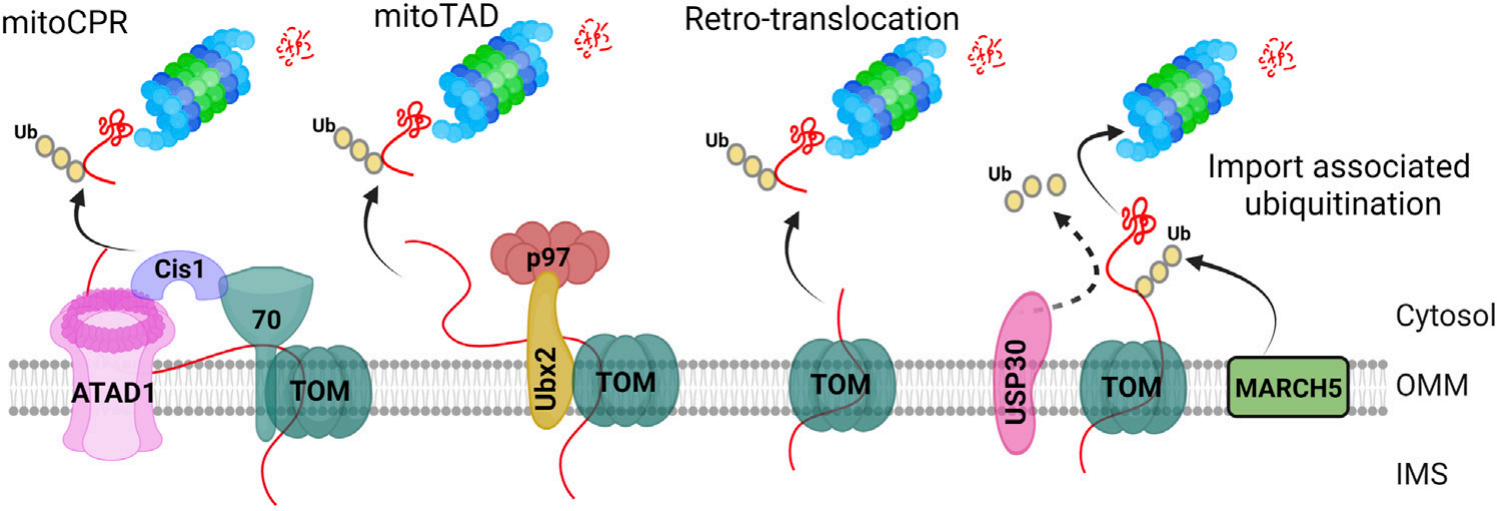
TOM complex associated ubiquitination of mitochondrial proteins. The “*mitoCPR*” pathway initiates through stress-stimulated dysfunctional mitochondrial protein import. It further causes localization of Cis1 at the TOM complex through its physical interaction with TOM70 subunit. Cis1 then recruits the AAA ATPase ATAD1/Msp1 at the TOM complex to remove the ubiquitinated protein and put them for proteasome mediated degradation. The “*MitoTAD*” pathway continuously checks the import channel, removing the blockade through TOM-Ubx2-p97/Cdc48 complex. The “*retrotranslocation*” pathway includes the export of unfolded intra-mitochondrial proteins to the cytoplasm for their ubiquitin-mediated proteasomal degradation. The “*import associated ubiquitination*” is mediated by OMM localized E3 ligase, March5, which constitutively ubiquitinates the incoming pre-proteins whereas USP30 deubiquitinase remove the ubiquitin, mediating mitochondrial protein import.

PTMs of TOM subunits play a crucial role in quality control. For instance, during mitochondrial stress, ubiquitylation of Tom22 can lead to mitochondrial membrane depolarization consequently (Wu et al., 2018). Additionally, the phosphorylation of Tom22 by casein kinase 2 (CSNK2) has been found to be essential for TOM complex biogenesis in yeast (Kravic et al., 2018). In contrast, in mammalian cells, phosphorylation of Tom22 is required for regulating Pink1/Parkin-related mitophagy, as it enhances Pink1 import. The loss of this phosphorylation impedes Pink1 import and promotes mitophagy (Horie et al., 2003). Furthermore, Tom70 undergoes ubiquitylation and phosphorylation as PTMs, but their precise functions remain to be fully explored (Zhang et al., 2017). To ensure the proper functioning of the TOM complex, the mitochondrial protein translocation-associated degradation (mitoTAD) pathway plays a critical role in quality control at the entry gate of the OMM. This pathway aims to prevent blocking of the TOM complex and retro-translocate IMS and IMM proteins through TOM complex into the cytoplasm for ubiquitin-mediated proteasomal degradation (Figure 3) (Ravanelli et al., 2020). To initiate the mitoTAD pathway, first Ubx2 binds to the TOM complex. Secondly, the cytosolic AAA ATPase Cdc48 then binds to the UBX domain of Ubx2 and pulls the substrate. Thirdly, it further causes the extraction and unfolding of the bound protein at the expense of ATP hydrolysis, leading to proteasomal degradation of the unbound protein (Bodnar and Rapoport, 2017). This process blocks the fusion of damaged mitochondria and accelerates their removal by mitophagy (Onishi et al., 2021). When mitochondrial proteins are stalled in the TOM complex due to defects in mitochondrial import, the Cis1-Msp1 recruitment mechanism is activated by the mitochondrial compromised protein import response (mitoCPR). Cis1, a cytosolic protein, is induced and localized to the OMM, where it connects AAA ATPase Msp1 and Tom70. Msp1 removes precursors from the import channel for proteasomal degradation (Figure 3).

In addition, the accumulation of Pink1 due to mitochondrial dysfunctions leads to interactions with various components of the TOM complex, such as Tom22, and Tom70 has been found to be critical for maintaining mitochondrial quality control (Lazarou et al., 2012; Maruszczak et al., 2022). The Tom70 and Parkin interaction triggers mitophagy upon mitochondrial import blockage, and Parkin mutant cells exhibit reduced Tom70-Parkin. Furthermore, Tom70 is important for Pink1 import (Kato et al., 2013), although further studies are required to understand the mechanistic insights on Pink1/Parkin import with the TOM complex.

**TABLE 1:** PTMs of the mitochondrial channels/transpoters

Name of the transporter/channel	Location	PTMs	References
TOM	OMM	Phosphorylation and ubiquitylation	Kravic et al. (2018); Zhang et al. (2017)
MIM	OMM	Phosphorylation	Schmidt et al. (2011)
VDAC	OMM	Acetylation, phosphorylation, GlcNAcetylation, ubiquitination and deamidation	Reina et al. (2016); Queralt-Martín et al. (2020); De Pinto et al. (2016); Johnsen et al. (2013); Pittalà et al. (2020)
ANT	IMM	Phosphorylation, acetylation, succinylation, glutathionylation and oxidation	Lam et al. (2013); Nguyen et al. (2013); West et al. (2006); Lozano et al. (2020)
TIM	IMM	Oxidation	Ramesh et al. (2016)
LETM1	IMM	Phosphorylation, and methylation	Huang et al. (2017); Liu et al. (2019)
MCU	IMM	Methylation, oxidation, phosphorylation, glutathionylation	Lee et al. (2016); Dong et al. (2017); Joiner et al. (2012); Zhao et al. (2019)
CLIC	OMM and IMM	Phosphorylation	Guo et al. (2018)
PiC	IMM	Methylation, phosphorylation and acetylation	Alves-Figueiredo et al. (2021)
mitoKCa	IMM	Phosphorylation, nitrosylation, and sulfhydration	Walewska et al. (2018); Rotko et al. (2020a); Walewska et al. (2022)
UCP	IMM	Ubiqutination, sulfonylation, glutathionylation	Mookerjee and Brand. (2011), Chouchani et al. (2016); Mailloux et al. (2011)

##### 2.1.1.2 Mitochondrial import (MIM) complex

MIM complex is also one of the major translocases to insert the proteins having a single or multiple α-helical membrane anchors. It is made up of two membrane-spanning alpha-helical proteins, Mim1 and Mim2, with Mim1 as a major constituent (Dimmer et al., 2012). Mim1 can form pores in a lipid bilayer, while Mim2 may recognizes positively charged residues in precursor proteins (Krüger et al., 2017).

The MIM complex is a highly dynamic system that operates in three distinct states or pools to import various precursor proteins into the OMM. In the first pool, the MIM-TOM complex accepts precursor proteins from Tom70. Tom70 imports single-spanning proteins such as Pth2 and Mcy1, while Tom20 helps import Gem1. Once these proteins are brought to the MIM complex, they are incorporated into the OMM. The second pool, known as the MIM-SAM complex, plays a crucial role in enhancing the early assembly events of small TOM subunits. The third pool, free MIM, inserts single-spanning proteins like Msp1 and Tom20 that are imported in a Tom70-independent manner. This dynamic coupling to partner proteins allows the MIM complex to import various types of precursor proteins, making it an essential regulator in the biogenesis of OMM proteins (Doan et al., 2020; Vitali et al., 2020). The MIM complex comprises multiple copies of Mim1, which form a channel in a lipid membrane, and one or two copies of Mim2 (Krüger et al., 2017). Recent studies have shed light on the critical role of the MIM complex in facilitating the import, assembly, and localization of OMM proteins (Vitali et al., 2020). By working together with TOM receptors and the SAM complex in a sequential and co-operative manner, the MIM complex can integrate different types of proteins into the OMM, including signal-anchored, tail-anchored, and single-spanning proteins. Nevertheless, further research is required to fully understand the molecular mechanisms by which sorting, and insertion of alpha-helical outer membrane proteins occur.

MIM is also involved in the biogenesis of various OMM proteins, such as Tom20, Tom70, and tail-anchored receptors Tom5, Tom6, and Tom7 (Thornton et al., 2010). So far, PTMs of MIM are not well studied. However, import of Tom70 precursor depends on phosphorylation at Ser12/16 of MIM1 by casein kinase 2 in Yeast (Schmidt et al., 2011). The cytosolic domains of Atg32 and Gem1, which are targeted to and integrated into the OMM, depend on MIM involvement, indicating its critical role in OMM protein biogenesis (Vitali et al., 2020). Moreover, MIM has been found to regulate the assembly of Fzo1 and Ugo1, two proteins involved in mitochondrial fusion (Dimmer et al., 2012), as well as Ubx2, an ER-resident protein involved in the ER-associated degradation pathway (Mårtensson et al., 2019), ultimately contributing to MPQC. The beta-barrel precursor proteins initially cross the TOM channel and are then incorporated via the SAM complex from IMS. The TOM receptors also bind to alpha-helical precursor proteins and bring them to the MIM complex. The MIM and SAM complexes work together to assemble the alpha-helical embedded and beta-barrel protein subunits of the TOM complex, highlighting the crucial role of the MIM complex in OMM protein biogenesis.

##### 2.1.1.3 Sorting and Assembly Machinery (SAM)

The sorting and assembly of complex β-barrel proteins in OMM requires additional machinery beyond the TOM complex, such as the SAM complex. The SAM complex mainly contains three subunits Sam50 (Tob55, Omp85), and the two peripheral components, Sam35 (Tom38, Tob38), and Sam37 (Mas37). Homology studies identified that human Sam50 is essential for the biogenesis of human Tom40 which is a β-barrel protein and Sam37 stabilizes mature Tom40 protein through electrostatic interactions, consequently facilitating successive TOM assembly (Humphries et al., 2005; Wang et al., 2021). Sam50 plays a key role in β-barrel precursor recognition and binding, as well as interacting with the MICOS complex for respiratory complex assembly (Ding et al., 2015). Additionally, Sam50 is involved in PINK1-Parkin-mediated mitophagy and mitochondrial dynamics, and recent research suggests its cooperation with p62/SQSTM1 mediates efficient mitophagy (Abudu et al., 2021). Sam37 is involved in the release of precursor proteins from SAM, indicating SAM’s substrate specificity is not limited to β-barrel proteins but also involves most α-helical Tom proteins. In *C. elegans*, the Sam50 homolog is essential for mitochondrial maintenance (Jian et al., 2018), suggesting its importance in mitochondrial function is evolutionarily conserved.

#### 2.1.2 Transport of metabolite across OMM

Being the most abundant key protein of the OMM, voltage-dependent anion channels (VDAC) is a β-barrel membrane protein that spans the inner and outer environment of OMM where it participates in and regulates most of the metabolites such as inorganic phosphates, ADP, pyruvate and certain amino acids (approximately 4 kDa of molecular weight) transported across the OMM based on their conductance property (Colombini, 2012; De Pinto, 2021; Mannella, 2021). It has two different conductance states, permitting a selective route for metabolites and ions: an open (anion selective high conductance) and a closed (slightly cation-selective low conductance). Regardless of little variation in the conformation states of the N-terminus, all possible structures were demonstrated to correspond to the open high conductance state whereas no structural explanation of the closed state has yet been evidenced (Choudhary et al., 2014). This different conductance is important for maintaining the membrane permeability of ATP/ADP, Ca^2+^ homeostasis, and apoptotic signaling. The dissociation of hexokinase from VDAC triggers apoptosis due to influx of Ca^2+^ into mitochondria (Keinan et al., 2010). Three VDAC isoforms, namely, VDAC1, VDAC2 & VDAC3 in humans have been found. VDAC2 and VDAC3 structures are less studied, however, all VDACs are believed to contribute to the same general structural features (Böhm et al., 2020). Although isoforms of VDAC are characterized by slight differences in their amino acid sequences, studies suggest different function in physiological and disease conditions for each VDAC isoform (Anflous et al., 2001; Cheng et al., 2003; Menzel et al., 2009).

VDAC undertakes various PTMs, namely, oxidative PTMs (which modify the redox status of a cell), acetylation, phosphorylation, GlcNAcetylation and ubiquitination. Different numbers of cysteines found in VDAC isoforms led researchers to study their PTMs. Mutagenesis studies in Yeast with the individual or cluster cysteines of VDAC3 showed an inverse relationship between the number of cysteines and the pore-forming activity, likely due to the formation of intrachain disulfide bridges, signifying PTMs role in modulating VDAC3 (Reina et al., 2016; Queralt-Martín et al., 2020). The study by the Saletti group revealed that VDAC cysteine residues can undergo progressive oxidation of sulfur which may be reversible or irreversible under physiological conditions. This oxidation consists of a series of events from redox reactive thiol (–SH) to disulfide bonds (RS-SR), sulfinic acid (SO_2_H), or sulfonic acid (SO3H), sulfhydration (SSH), and sulfenylation (SOH) (Reina et al., 2020). Oxidative PTMs of cysteines suggested a preferential sensitivity to oxidation for each sulfur-containing amino acid of VDAC (De Pinto et al., 2016). A distinct modification of each cysteine can correlate to a specific functional/structural role of VDAC, understanding the oxidative potential for reactive oxygen species (ROS) generation, especially in IMS which has acidic pH and is favorable for oxidation. Other common PTMs such as phosphorylation at Ser104 by a protein kinase C (PKC), a cAMP-dependent protein kinase A (PKA), and a glycogen synthase kinase-3β (GSK3β) and acetylation at the N-terminal amino acid have also been found in VDACs. PKA and c-Jun N-terminal kinase-3 (JNK3) mediated phosphorylation of VDAC1 controls apoptotic activity, modulating its interaction with cytoskeletal components (Kerner et al., 2012; Reina et al., 2020). Elevated levels of VDAC O-GlcNAcylation are seen in rats having low running capacity, indicating this modification may serve increased mitochondrial stability (Johnsen et al., 2013). Moreover, the extent of ubiquitination of VDAC3 is observed directly proportional to the duration of hypothermia, may serving as a protective pathway in hypothermia against oxygen-glucose deprivation/recovery (OGD/R) (Zhao et al., 2020). Furthermore, a very unusual PTM, the deamidation at asparagine (Asn37, Asn106, Asn207, Asn214, and Asn239) and glutamine (Gln166 and Gln226) of VDAC1 was discovered in response to oxidative stress and known to interact with superoxide dismutase1 (SOD1) mutant (SOD1G93A) in NSC34 cells of amyotrophic lateral sclerosis (ALS) (Pittalà et al., 2020). The structural changes in VDAC1 due to these PTMs clarifies the interaction between VDAC1 and mutant form of SOD1 as well as ALS pathogenesis (Pittalà et al., 2020). Although the evolutionary roots highlight the importance of VDACs in mitochondria, gaining a deeper understanding of the connection between the structure and function of VDAC conductance, the structural variances influenced by the placement of PTMs in amino acid sequences, and their voltage-dependent mechanism would simplify the investigation of VDAC isoforms' functions and their involvement in neurodegenerative therapeutics.

Not all three isoforms of VDAC but VDAC1 is known to have a function in MPQC. An OMM localized translocator protein, TSPO was found to interact with VDAC1, stimulating a ROS-mediated inhibition of activation of PINK1/Parkin-dependent mitophagy (Gatliff et al., 2014). In Pink1/Parkin-dependent mitophagy, depolarization of the IMM results in the accumulation of Pink1 on the OMM, recruiting Parkin, an ubiquitin ligase. This in turn leads to the ubiquitination of the OMM proteins, including VDAC, and consequent degradation by the proteasome. Under TSPO-VDAC1 interaction, increased ROS levels cause a change in ΔΨm that enables Pink1 kinase import and blocks recruitment of Parkin and thereby mitophagy. TSPO overexpressing cancerous cells caused increased VDAC1 binding that led to dysfunctional mitochondria, signifying that coordinated TSPO-VDAC1 interaction is required to maintain mitochondrial homeostasis (Shoshan-Barmatz et al., 2019). Further information is needed on the structure of the closed state, how binding partners or ΔΨm could lead to the open/closed states, the function and mobility of the N-terminal α-helical domain of VDAC, and the physiological role of VDAC oligomers.

#### 2.1.3 OMM channels involved in cell death

Mitochondrial Apoptosis-induced Channel (MAC) is a voltage-independent and gigantic channel having high conductance for both heterogeneous and small proteins like cytochrome c and is regulated by Bcl-2 family proteins. Nonetheless, MAC and Permeability Transition Pore (PTP) opening may behave alone or accompanied, based on cell type and death stimuli (Pavlov et al., 2001). Moreover, MAC activity is considerably unique from the VDAC and TOM complex which are integral channels of OMM. The very basic function of MAC is to provide the passage for the release of cytochrome c (12.5 kDa) in early apoptotic events. The onset of MAC activity corresponds to the release of cytochrome c in several cell systems (Martinez-Caballero et al., 2009). Bcl-2 family members are responsible for MAC formation and its activity. The loss of MAC activity and oligomeric Bax (pro-apoptotic studies) provide strong data that oligomeric activated Bax is a structural component of MAC (Dejean et al., 2005). On the contrary, anti-apoptotic molecules like Bcl-2 prevent Bax oligomerization, directly binding to pro-apoptotic effectors (Leber et al., 2007). Regulation of MAC is also mediated through BH-3-only proteins. These proteins are required to decipher the survival and death signals throughout the cell. They act either as activators or sensitizers. Bid, Bim, p53, and perhaps PUMA directly interact with Bax and Bak, causing a conformational change, which activates their oligomerization leading to MAC formation (Chipuk and Green, 2008).

MAC directly is not involved in the modulation of MPQC. However, Bcl-2 family members were found to take part in the maintenance of a healthy mitochondrial milieu, regulating Parkin/PINK1-dependent mitophagy. Bcl-xL and Mcl-1 bind to Parkin/PINK1 and prevent Parkin translocation to depolarized mitochondria and thus block Parkin-dependent ubiquitination of mitochondrial substrates and downstream signaling, implying Bcl-2 effectors can also be a target for therapeutics (Hollville et al., 2014; Zhang et al., 2020).

#### 2.1.4 OMM as a site of inter-organelle contacts and lipid transfer

Mitochondria–Endoplasmic reticulum contact sites (MERCs) [Endoplasmic reticulum–mitochondria encounter structure (ERMES) in yeast] is a multimeric protein complex that joins the endoplasmic reticulum (ER) and OMM. The tethering proteins from both ER and mitochondria build a bridge to form MERCs. The tethering molecules include vesicle-associated membrane protein associated protein B (VAPB) (De Vos et al., 2012), protein tyrosine phosphatase-interacting protein-51 (PTPIP51) (De Vos et al., 2012), mitofusin1/2 (MFN1/2) (Sebastián et al., 2012), inositol 1,4,5-trisphosphate receptor (IP3R)-glucose-regulated protein 75 (GPR75)-voltage-dependent anion channel (VDAC1) complex (Szabadkai et al., 2006a), and B-cell receptor-associated protein 31 (BAP31) and fission 1 homolog (FIS1) (Iwasawa et al., 2011). MERCs are notable to shape the flux of Ca^2+^ and to maintain synthesis and composition of membrane lipids, shuttling import and export of lipids (Petrungaro and Kornmann, 2019). MERCs are enriched with two transmembrane proteins; Sarco/endoplasmic reticulum Ca^2+^ ATPase (SERCA) and inositol 1,4,5-trisphosphate receptors (IP3R) provide Ca^2+^ shuttle service from the cytosol into the ER lumen (Camello et al., 2002) and Ca^2+^ from the ER lumen into the mitochondria (Szabadkai et al., 2006b). The ER-mitochondrial resident structural proteins such as GTPase MFN2 also participate in Ca^2+^ signaling (Naon et al., 2016). It directly binds and transfers phosphatidyl serine (PS) across the ER-mitochondria juncture (Hernández-Alvarez et al., 2019) to convert it into phosphatidylethanolamine (PE) (Ardail et al., 1991). PS to PE conversion warrants the suitable environment for PS transport and mitochondrial health (Horvath and Daum, 2013). Apart from Ca^2+^ signaling, homeostasis of membrane lipids is one of the signatory roles of MERCs. The precursor of cardiolipin (CL) synthesis, phosphatidic acid mostly comes from the ER through MERCs (Potting et al., 2013) and CL is very well known to be involved in various mitochondrial physiological processes like mitochondrial bioenergetics, structure, and mitophagy (Hsu et al., 2015). Besides CL, MERCs also serves as site for phosphatidyl serine and other lipid synthesis pathways. Lipid droplets bud from ER and provide fatty acids to mitochondria for regulating fatty acid oxidation and the tricarboxylic acid (TCA) cycle. The abundance of cholesteryl esters (CE) and triacylglycerols (TG) producing enzymes; acyl-CoA cholesterol acyltransferase-1 (ACAT1) and diacylglycerol acyltransferase 2 (DGAT2) respectively at MERCS implies their role in the biogenesis of lipid droplets (Stone and Vance, 2000; Stone et al., 2009). Another entity of ER-mitochondria tethering proteins, PDZ domain-containing protein 8 (PDZD8) ER transmembrane protein is recognized as a regulator of Ca^2+^ dynamics in neurons (Hirabayashi et al., 2017).

In yeast, ERMES is mainly formed by the mitochondrial distribution, and morphology (MDM) complex. The OMM protein, mitochondrial distribution, and morphology protein 10 (Mdm10) is a subunit of both MDM and SAM complexes, physically connecting the OMM and ER membrane to help in lipid transport between the two membranes (Esposito et al., 2019). The switching between MDM and SAM is controlled by Tom7. Thus, these interlinking TOM, SAM, and ERMES complexes behave as a functional network, maintaining mitochondrial structure and the transport of metabolites. Along with Mdm10, other OMM proteins like Mdm12, Mmm1, and Mmm2 are also components of ERMES. Moreover, other transporters, like VDAC, Tom70, etc., are also involved in the formation of ER-mitochondria contact sites (Meisinger et al., 2006; Kornmann et al., 2009; Murley et al., 2015). The SAM-Mdm10 complex offers an assembly platform for the TOM complex and the Tom22 precursor uses Mdm10 for its biogenesis, fostering an effective assembly of the TOM complex. Interestingly, ERMES is connected to SAM via the Mdm10, and ERMES subunits show strong genetic interactions with mitochondrial contact site and cristae organizing system (MICOS) subunits to maintain mitochondrial organization (Hoppins et al., 2011). Give and take relationship between ER and mitochondria provides a platform to understand the lipid metabolism and signaling in both ER and mitochondria compartments, extending the crucial role of MERCs in diseases linked with alterations in lipid pool.

### 2.2 IMM channels, their control mechanisms, and PTMs

IMM is the second level of entry barrier which is highly selective and more secure for entry into the MM. The transporters or ion channels, the part and parcel of IMM, are keenly engaged in maintaining mitochondrial processes.

#### 2.2.1 ADP, ATP translocase (ANT)

ANT is an antiporter in nature and belongs to the mitochondrial carrier family (SLC25). It exchanges ATP from the MM and ADP from ATP consumption in the remainder of the cell. The ANT can be found in two states; the matrix and the cytoplasmic states, compatible with the establishment of a high-conductance carrier to dissipate the ΔΨm and lead to mitochondrial swelling (Vieira et al., 2000). Initially, it was supposed as a dimer, but structural, functional, and biophysical data now prove that this exists and works as a monomer (Kunji and Ruprecht, 2020). Other than ADP/ATP exchange, it also involves in stimulation of the Mitochondrial Permeability transition Pore Complex (MPTP) by Ca^2+^ and ROS, a mild uncoupling movement that is activated by anion superoxide, peroxidized lipids, and fatty acids. All these functions are controlled by ΔΨm (Brenner et al., 2011). ANT also modulates mitophagy, the coordinated degradation of mitochondria, irrespective of its nucleotide translocase catalytic activity. In response to the bioenergetic collapse due to mitochondrial stress causing agents (either CCCP or suppressors of OXPHOS), the ANT complex is necessary for inhibition of the presequence translocase TIM23, stabilizing PINK1. ANT controls TIM23 indirectly, interacting with TIM44. ANT1 knockout mice led to blunted mitophagy, accumulating aberrant mitochondria, and indicating a novel function of ANT as a fundamental facilitator of mitophagy (Hoshino et al., 2019).

ANT undergoes numerous PTMs such as phosphorylation, acetylation, succinylation at lysine, *etc.* ANT phosphorylation at T84 and Y191 in the hearts of mice and humans have been observed vital for cell fate and heart conductance (Lam et al., 2013). Acetylation of ANT is associated with compromised cardiac metabolism in mitochondria in the absence of Cyclophilin D (CypD) in mice (Nguyen et al., 2013). Moreover, ANT S-glutathionylation has been found on Cys57 in cardiac-specific inducible NO synthase transgenic mice (Finoshin et al.). The CypD binding site is situated alongside the glutathiolated Cys57, modulating its function (West et al., 2006). Oxidation of the thiol group of ANT is recently noticed to lead to mitochondrial dysfunction through MPTP opening upon silicon dioxide (SiO_2_) nanoparticles treatment (Lozano et al., 2020). The structural aspects of the conformational changes for the interconversion of matrix and cytoplasmic states and the free and substrate-bound intermediates still ought to be solved. Further functional investigations are necessary to find out PTMs, deepening insights into molecular mechanisms and physiological relevance in diseases.

#### 2.2.2 Translocase of the inner membrane (TIM)

TIM is the major gateway of IMM. TIM23, translocase of IMM comprises Tim17, Tim21, Tim23, and Tim50 proteins which are incorporated into the IMM displaying functional domains to the IMS side, and Tim44, Tim14/Pam18, Tim16/Pam16, Pam17, and Tim44 facing matrix side. Tim17 shares sequence similarity with Tim23 presumably to form the translocation pore in the IMM. Tim50 guides preproteins released from TOM to enter the TIM23 complex and this transport is further controlled by Tim21. The small TIM chaperone molecules are imported to the OMM by an N-terminal end of Tom40. Tim54 interacts with small TIM chaperones through its IMS domain and sends the precursors to the carrier translocase of the inner membrane (TIM22) in a ΔΨm-dependent manner to release the proteins laterally into IM(Herrmann and Neupert, 2013). The translocation from TOM to TIM is regulated by the membrane potential, bolstering the passage of the positively charged pre-sequences to the negatively charged matrix side of IMM. The mitochondrial HSP70 chaperone protein (mtHSP70) then forms a complex with the pre-sequence associated motor (Hernández-Alvarez et al.), interacting with TIM23 core components to force the proteins inside the matrix at the expense of ATP. TIM23 also interacts with complexes III and IV to translocate pre-protein into the matrix in an ATP-driven manner. Mostly, the pre-sequence is cleaved by matrix mitochondrial processing peptidase (MPP). The TIM23 complex also confirms the lateral insertion of precursor proteins into the IMM(Schulz et al., 2011). The mitochondrial intermembrane space import and assembly (MIA) machinery brings in cysteine-rich proteins such as the small TIM proteins into the IMS for their oxidative folding. In addition to this, TOM complex precursors are also guided by TIM to SAM complex. The oxidase assembly (OXA) translocase mediates the sorting of IMM proteins imported by TOM and Tim23-PAM machinery and exports them to IMM. Interestingly, the N terminus of Pink1 is also imported through the Tim23 import pathway (Silvestri et al., 2005), proposing the possible role of TIM23 in mitophagy.

Although PTMs of TIMs are poorly understood, it has been studied that oxidation of Tim17 by sulfhydryl oxidase Erv1 is mediated by the mitochondrial disulfide relay. Tim17 contains a pair of highly conserved cysteine residues which form a disulfide bond when it is exposed to the IMS. This disulfide bond mediates efficient protein translocation through the TIM23 complex and dynamic gating of its pre-protein-conducting channel (Ramesh et al., 2016).

#### 2.2.3 Ion exchangers

Mitochondrial calcium (_m_Ca^2+^) plays an essential role in many cellular functions maintaining proper levels of both cytosolic and mitochondrial Ca^2+^ which is critical for cellular homeostasis. _m_Ca^2+^ efflux is regulated by both Na^+^-dependent (mitochondrial Na^+^–Ca^2+^ exchanger, NCLX) pathway and an Na^+^-independent (H^+^–Ca^2+^ exchanger) pathway. NCLX is a distinct exchanger from the plasma membrane Na^+^–Ca^2+^ exchanger (NCX) and is found in most cell types and tissues, with activity mainly observed in excitable cells (Jiang et al., 2009; Palty et al., 2010). The activity of the H^+^–Ca^2+^ exchanger is mainly found in non-excitable cells. The ΔΨm, stoichiometry of NCLX, and electrochemical gradient for Na^+^ entry into the MM from the cytosol all influence Ca^2+^ extrusion from the matrix (Jiang et al., 2009). NCLX is endogenously expressed in the IMM (Palty et al., 2010), consisting of two transmembrane domains, α1 and α2, which bind ions and translocate them across the membrane (Khananshvili, 1991). The regulation of NCLX by ΔΨm is mediated through catalytic and regulatory domains, which can link mitochondrial metabolic state and Ca^2+^ signaling (Kostic et al., 2015). NCLX is also post-translationally regulated by phosphorylation at Ser258 by protein kinase A (Yang et al., 2003; Kostic et al., 2015) and palmitoylation at cysteine (Reilly et al., 2015), which ubiquitously regulates Ca^2+^ homeostasis and ΔΨm in cells.

HCX (H^+^/Ca^2+^): HCX activity is shown to be dominant in certain tissues such as lung, liver, smooth muscles, and kidney where NCLX activity is weak (Gunter and Pfeiffer, 1990). Though the molecular identity of HCX is yet to be revealed, high-throughput RNA interference screening in fruit fly, *Drosophila* identified a gene responsible for mitochondrial Ca^2+^ and H^+^ [LETM1 (leucine–zipper–EF hand-containing transmembrane region), the human homolog] (Jiang et al., 2009). Initial studies suggested that LETM1 functions as a Ca^2+^ influx candidate (Jiang et al., 2009) but later studies demonstrated Ca^2+^ efflux from cardiac mitochondria in a free matrix Ca^2+^ concentration dependent manner (Natarajan et al., 2020). The function of LETM1 is controversial and debated as to whether it acts a K^+^/H^+^ exchanger (KHE) as originally supposed or an Ca^2+^/H^+^ exchanger (CHE) as evidenced in studies (Austin and Nowikovsky, 2019; Lin and Stathopulos, 2019). LETM1-KD H9c2 cells altered Ca^2+^ efflux, indicating LETM1-HCX mediated Ca^2+^ efflux (Natarajan et al., 2020) whereas another finding showed that LETM1 mediated mitochondrial Ca^2+^ regulation is facilitated by a LETM1-KHE activity, modulating the activity of the mitochondrial Na^+^/H^+^ exchanger (mNHE) eventually affecting NCLX-mediated Ca^2+^ efflux (Austin et al., 2017). A recent study demonstrates that MICS1 is the long-sought mitochondrial CHE and interacts with LETM1, modulating its activity (Austin et al., 2021). Irrespective of its individuality, LETM1 has been implicated in interaction with signaling proteins such as MRPL36, BCS1L and CTMP to modulate protein import and assembly in IMM, mitochondrial morphology and metabolism (Park et al., 2014; Li et al., 2019).

LETM1 is known to be a direct target for phosphorylation by PINK1, which constitutively phosphorylates LETM1 and regulates Ca^2+^ signaling through a LETM1-CHE activity (Huang et al., 2017). LETM1 methylation has also been identified as an epigenetic marker in obese patients associated with fasting insulin levels (Liu et al., 2019). While LETM1’s primary role as HCX or CHE is still unclear, loss of LETM1 has been shown to adversely affect mitochondrial Ca^2+^ signaling.

In addition to LETM1, Transmembrane BAX Inhibitor-1 Motif 5 (TMBIM5) has been recently investigated as a Ca^2+^/H^+^ exchanger in the IMM that allows Ca^2+^ efflux from mitochondria, preventing mitochondrial membrane hyperpolarization (Austin et al., 2022; Patron et al., 2022). *In vivo* and *in vitro* gene alteration studies show that TMBIM5 is an essential unit of mitochondrial ion transport. Overexpression of TMBIM5 carries out mitochondrial Ca^2+^ uptake through aspartate residue of channel pore whereas TMBIM5 loss modulates matrix ion composition with no major effect on Ca^2+^ uptake (Zhang et al., 2022). Further studies are required to explore the mechanism of mitochondrial ion homeostasis by both TMBIM5 and LETM1.

#### 2.2.4 Mitochondrial calcium uniporter channel (mtCU/MCU)

_m_Ca^2+^ is a versatile molecule that regulates intracellular signaling events under both physiological and pathological conditions. The fundamental physiological role of the mitochondrial calcium uniporter (MCU) is to modulate the Ca^2+^ signaling, aerobic metabolism as well as apoptosis (De Stefani et al., 2015). However, excessive Ca^2+^ accumulation in the MM can trigger cell death through the opening of the MPTP. Therefore, maintaining the balance of Ca^2+^ levels is essential, which is achieved through the activity of IMM Ca^2+^ permeable transporters and channels (Zavodnik, 2016). MCU is a conserved protein found throughout eukaryotes, except in yeast. MCU is primary route for the Ca^2+^ influx in MM and only known channel to allow acute Ca^2+^ entry in the MM (Palty et al., 2010; Baughman et al., 2011). MCU’s function has been well documented in cellular proliferation and apoptosis (Bachmann et al., 2019). This multi-subunit complex consists of MCU (pore-forming protein), MCUB (an MCU paralog), EMRE (essential MCU regulator), the regulatory Ca^2+^ sensing MICU proteins (MICU1, MICU2 & MICU3), and MCUR1 (mitochondrial calcium uniport regulator 1) (Perocchi et al., 2010; Baughman et al., 2011; De Stefani et al., 2011; Mallilankaraman et al., 2012a; Mallilankaraman et al., 2012b; Plovanich et al., 2013; Raffaello et al., 2013; Sancak et al., 2013; Tomar et al., 2016; Patron et al., 2019). EMRE and MCUR1 act as scaffolding regulators of MCU channel and essential for _m_Ca^2+^ flux. MCUR1 directly interacts with MCU and enhances Ca^2+^ uptake inside mitochondria (Vais et al., 2015). Downregulation of MCUR1 reduces mitochondrial Ca^2+^ uptake, OXPHOS activity, and hence ATP production (Mallilankaraman et al., 2012b; Tomar et al., 2016). The cytosolic Ca^2+^ threshold is set by MICU1, if the level of Ca^2+^ is exceeded above the threshold, i.e. 1-2 μM in most cell types, the EF-hands of MICU1 sense this elevation and switch the status of the channel from closed to open conformation (Mallilankaraman et al., 2012b).

The activity and expression of MCU and its regulators are regulated at several levels, transcriptional, post-transcriptional, and post-translational levels. Small non-coding regulatory microRNAs, such as miR-25 and miR-138, regulate the expression of MCU subunits (Hong et al., 2017). The majority of PTMs of MCU include methylation, oxidation, and phosphorylation. Oxidation of conserved cysteines, including C67, C97, and C191, in the N-terminal domain (NTD) by increased levels of ROS leads to S-glutathionylation of C97 (Dong et al., 2017). This modification remodels the conformational change in NTD of MCU, boosting MCU activity, and increasing the rate of Ca^2+^ entry into the MM (Lee et al., 2016; Dong et al., 2017). Another PTM is phosphorylation by Calmodulin kinase II (CaMKII) at S57 and S92 of the NTD of the MCU complex (Joiner et al., 2012) as well as phosphorylation by the proline-rich tyrosine kinase 2 (Pyk2) and AMP-activated protein kinase (AMPK). Both Pyk2 and AMPK-dependent MCU phosphorylation enhances Ca^2+^ entry to the matrix and intensifies mitochondrial respiration and energy production (Zhao et al., 2019).

Moreover, the MCU regulator, MICU1 is modulated through arginine methyltransferase 1 (PRMT1), reducing Ca^2+^ uptake into the matrix (Madreiter-Sokolowski et al., 2016). Recent studies have also shown that the S92 of MCU NTD is phosphorylated by protein kinase C isoforms, namely, PKCβII, PKCδ, and PKCε, which amazingly switch the exchange between cytosolic Ca^2+^ and mitochondrial Ca^2+^ and manipulate the expression level of MCU (Lee et al., 2020). Moreover, MCU, previously known solely as a Ca^2+^ transporter, has been found to play a role in mitochondrial quality control. Cho and colleagues demonstrated that during mitochondrial fission, the recruitment of Drp1 occurs at regions where the ΔΨm is reduced. At this point, Drp1 interacts with the mitochondrial zinc transporter Zip1-MCU complex, allowing for Zn^2+^ entry and subsequent fission. Healthy mitochondria are able to restore MMP levels through the fusion-fission cycle, while dysfunctional mitochondria undergo mitophagy (Cho et al., 2019). Additionally, MCU upregulation has been shown to activate calpain and reduce OPA1 levels during ischemia/reperfusion (IR) injury, resulting in an imbalance in mitochondrial dynamics (Guan et al., 2019). However, further research is needed to fully understand the molecular mechanisms by which MCU contributes to mitochondrial quality surveillance.

#### 2.2.5 Mitochondrial chloride (Cl^−^) channel proteins

Chloride channels (Cl^−^) are a diverse group of proteins predominantly present in intracellular organelles, serving important roles in many physiological processes. The different types of Cl-channels include the voltage-sensitive ClC subfamily, glycine receptors, transmitter-gated GABAA, Ca^2+^-activated Cl-channel, the cystic fibrosis transmembrane conductance regulator (CFTR), volume-regulated channel, and high (maxi) conductance Cl-channel (Ashley, 2003). In addition, a few Chloride Intracellular Ion Channels (CLICs) have recently been shown to be localized to mitochondria. Two mitochondrial chlorides (mtCl) channels, namely, CLIC4 and CLIC5 have been identified in mitochondria (Ponnalagu and Singh, 2017). CLIC4 was first identified to be present on IMM of mouse keratinocyte mitochondria (Fernández-Salas et al., 1999) whereas in adult cardiomyocytes, it is found in OMM. CLIC5 is located in IMM of adult cardiomyocytes (Ponnalagu et al., 2016). These belong to the class of intracellular anion channels (CLICs) and have been shown to play a role in various physiological processes.

Recent studies have shown the presence of CLIC4 in mitochondrial-associated membranes, implicating its involvement in cardioprotection (Ponnalagu et al., 2022). There are six paralogs of CLICs (CLIC1-CLIC6) stated in mammals, four (AtDHAR1-AtDHAR4) in *Arabidopsis thaliana*, three in invertebrates in *Drosophila Melanogaster* (DmCLIC) and two in *Caenorhabditis elegans* (EXC4 and EXL1) (Gururaja Rao et al., 2017; Ponnalagu and Singh, 2017; Gururaja Rao et al., 2018). It is suggested to have a pro-apoptotic role in p53-mediated apoptosis (Fernández-Salas et al., 2002). The other chloride channel is from the ClC family, ClC-Nt, and has been intended as a component of IMAC (Lurin et al., 2000). Despite several articles on mtCl channels, understanding their molecular identity, structure, and role in physiological and pathological states is yet inadequate.

CLICs have also been found to be modulated by PTMs such as phosphorylation. Cyclin-dependent kinase 5 mediated serine phosphorylation of CLIC4 stimulates its stability and regulates cell death (Guo et al., 2018). In addition to CLICs, Maxi-Chloride channels are also present in mitochondria. While their molecular structure remains unidentified, it is believed that they have a strong relationship with the permeability transition pore complex (PTPC) as their activity is similar to that of monomeric PTPC (Sabirov et al., 2006). It is well known that CLICs modify mitochondrial activity through modulation of ROS levels. For example, the loss of CLIC5 in cardiac mitochondria has been shown to enhance mitochondrial ROS and modulate the Ca^2+^ retention capacity of mitochondria (Ponnalagu et al., 2016; Ponnalagu et al., 2019). However, the direct influence of mitochondrial CLICs on mitochondrial function remains unclear and requires further investigation. Overall, the role of mitochondrial chloride channels in regulating mitochondrial activities is complex and requires further research. A better understanding of the molecular identity, structure, and function of these channels in physiological and pathological states could potentially lead to the development of new therapeutic strategies for the treatment of various diseases.

#### 2.2.6 Mitochondrial inner membrane anion channel (IMAC)

The inner mitochondrial membrane (IMM) harbors various chloride (Cl-) anion-selective channels, including the inner membrane anion channel (IMAC). IMAC has a single-channel conductance of approximately 100 pS, with slight anion selectivity. Small molecules such as bicarbonate, phosphate, chloride, citrate, ATP, and succinate can pass through this channel. The modulation of IMAC by palmitoyl-CoA Mg^2+^, amphiphilic amines, and pH has also been observed. Although the function of IMAC in pathophysiological conditions is well established, its physiological role is still uncertain, as it appears to facilitate ion movement only in the presence of an alkaline matrix environment. It has been proposed that anion efflux through IMAC can be a protective mechanism against excessive matrix swelling (Beavis, 1992). Recently, mitochondrial anion efflux has been linked to hypoxic preconditioning, further highlighting the potential importance of IMAC in cellular homeostasis (Vanden Hoek et al., 1998). Despite its significance, the molecular identity of IMAC remains elusive, which is the major limitation in researching this channel. The regulation of IMAC activity has been linked to glutathione levels, where inhibition of mitochondrial glutathione uptake, the NADPH-dependent glutathione reductase, or the NADH/NADPH transhydrogenase was found to stimulate IMAC opening (Aon et al., 2007). These findings suggest that redox-dependent activities might play a role in modulating IMAC activity. Further studies are required to understand the molecular identity of IMAC and the underlying redox-dependent PTMs.

#### 2.2.7 Mitochondrial phosphate carrier (PiC)

The PiC is an important mitochondrial phosphate carrier protein, encoded by SLC25A3 in humans. It facilitates the transport of inorganic phosphate, a key substrate of OXPHOS, across the IMM. The mammalian mitochondrial PiC is ubiquitously expressed and exists in two distinct isoforms, PiC-A and PiC-B. Together with ANT and complex V, PiC forms the ATP synthase microdomain for ATP production. Furthermore, PiC transport is significant for efficient mitochondrial Ca^2+^ handling. _m_Ca^2+^ uptake requires the presence of anions such as Pi, acetate, bicarbonate, and glutamate to provide protons for the respiratory chain. Reduced anions restrict Ca^2+^ uptake, leading to low O2 consumption. Pi may be required for both boosting the MCU-mediated Ca^2+^ influx and inhibiting the exchanger-mediated Ca^2+^ efflux (Chalmers and Nicholls, 2003; Wei et al., 2012). PiC transgenic mice with elevated and shRNA mediated diminished levels of cardiac PiC have been characterized by cardiac hypertrophy, unaltered respiration, ATP content, Ca^2+^ retention, and activation of the MPTP (Gutiérrez-Aguilar et al., 2014). Alongside, inducible and cardiac-specific deletion of PiC also showed the same phenotype as seen in shRNA mediated deletion of PiC. Notably, time-dependent mitochondrial phosphate uptake was eliminated, and the mitochondrial ATP content was reduced with no impaired Ca^2+^ retention and was in fact elevated. However, the absence of phenotypes of the PiC transgenic mice might be due to the abundance of PiC which does not regulate the transport in accord with PiC’s high turnover rate. The diminished ATP and cardiac hypertrophy in PiC depleted model are in line with the phenotype of the ANT knockout mouse, in which another component of the ATP synthasome is absent (Narula et al., 2011). Hence, insufficient Pi transport and oxidative phosphorylation might be the reasons for PiC phenotypes. Pathogenic mutations in PiC show different phenotypes based on tissue-specific expression and distinctive enzymatic activity of isoforms. Three PTMs for PiC, namely, methylation at lysine 112, phosphorylations at tyrosine 196 and 246, and acetylation at lysine 99, 209, and 234 are associated with cardiac diseases (Alves-Figueiredo et al., 2021). More studies are warranted to uncover PiC mutations in various models in stress response.

#### 2.2.8 Potassium (K^+^) channels

Mitochondrial K^+^ channels play an important role in regulating the electrochemical gradient and mitochondrial function. Two types of K^+^ channels located at the IMM, mitoKATP and mitochondrial Ca^2+^ activated K^+^ channel (mitoKCa), are regulated by various factors including ROS, ATP, Ca^2+^ and free fatty acids (Wrzosek et al., 2020).

##### 2.2.8.1 Mitochondrial K_ATP_ (mitoK_ATP_)

MitoKATP was first observed in the liver and is similar to the KATP channel present in the sarcolemma of cardiac cells, albeit with a lower conductance. The elevated mitochondrial inner membrane permeability to K^+^ has been hypothesized to enhance tolerance to IR injury in a cell (O'Rourke, 2007). Recently, a novel protein complex consisting of a channel forming subunit (MITOK) and a regulatory subunit carrying the ATP-binding domain (MITOSUR) was identified for ATP-sensitive K^+^ transport across the IMM (Paggio et al., 2019). MitoKATP facilitates the uptake of potassium (K+) driven by the negative ΔΨm and is repressed by physiological ATP levels. Endogenous molecules such as adenosine and bradykinin can decrease infarct size by stimulating mitoKATP channels in a PKC-dependent approach.

##### 2.2.8.2 Mitochondrial KCa

KCa channel family members are categorized through their differing single channel conductance into three types, namely, big conductance (BKCa, 200–300 pS), intermediate conductance (IKCa, 32–39 pS) and small conductance (SKCa, 4–14 pS) channels (Diness et al., 2015). In addition to plasma membrane, KCa channels have also been localized in mitochondria. The large-conductance Ca^2+^ -activated K^+^ channel (BK channel) is also present in the IMM of cardiomyocytes and brain, suggesting that the molecular identity of mitoKCa could be similar to the BK channel. These channels take part in oxidative regulation at the plasma membrane. However, no study is done in the context of their redox-sensitive PTMs (Nishida et al., 2009). Therefore, elucidating the molecular basis of redox-sensitive PTMs in these channels could provide new insights into oxidative regulation at the plasma membrane. Another selective K^+^ channel detected in IMM from human glioma cells by direct patch-clamp of mitoplasts is the Ca^2+^-stimulated K^+^ channel (mitoKCa) (Siemen et al., 1999). _m_Ca^2+^ uptake can be raised through mitoKCa against extreme mitochondrial Ca^2+^ accumulation and that might control the tuning between mitochondrial volume and/or Ca^2+^ accumulation under increased cardiac workload conditions (O'Rourke, 2007). SKCa channels are shown to be localized in IMM of cardiomyocytes and neurons, regulating mitochondrial respiration and _m_Ca^2+^ uptake (Stowe et al., 2013; Honrath et al., 2017). MitoSK channel activation generates a protective decrease in ischaemia-induced mitochondrial Ca^2+^ overload and ROS, balancing sarcoplasmic reticular (Ponnalagu et al.) Ca^2+^ release (Stowe et al., 2013). These findings together imply SK2 channels as potential targets for ameliorating ROS mediated mitochondrial stress.

##### 2.2.8.3 Kv1.3

The Kv1.3 channel, which is identical to the plasma membrane Kv1.3 channel, has been detected in the IMM of Jurkat T lymphocytes (Szabò et al., 2005). This channel has increased expression in cancerous cells, making it a potential target for drug therapy (Szabò et al., 2005). However, the molecular identity and pharmacology of these channels are not well studied, and further research is necessary to develop treatments against diseases by targeting these channels. The experimental evidence on PTMs of mitoK channels is far limited. It has been found that the activity of mitoKATP and mitoBKCa is regulated by PKC, PKA and PKG through phosphorylation. The phosphorylation of mitochondrial potassium channels might induce cardioprotective cascade (Rotko et al., 2020b; Checchetto et al., 2021). mitoK channels are regulated by gaseous transmitters such as carbon monoxide (CO), hydrogen sulfide (H_2_S) or nitric oxide (NO) through PTMs such as S-nitrosylation or S-sulfhydration (Walewska et al., 2018; Rotko et al., 2020a). A very recent study showed that applications of the H_2_S donor NaHS causes mitoBKCa S-sulfhydration of cysteines without hampering the channel activity (Walewska et al., 2022).

#### 2.2.9 Uncoupling proteins (UCPs)

Uncoupling proteins (UCPs) are a group of mitochondrial transporter proteins that play a crucial role in the regulation of energy metabolism and redox homeostasis (Ponnalagu and Singh, 2017). In response to stress conditions, ROS overproduction and dysfunction in ATP synthesis, mitoK_ATP_ channel and UCP get activated and produce mild uncoupling. This in turn attenuates mitochondrial ROS formation (Zhang et al., 2001). This feedback-induced decreased ROS protects mitochondria and allows for rapid oxidation of the reducing equivalents that overfeed the mitochondrial respiratory chain. However, under physiological conditions, the mitoK_ATP_ channel and UCPs are likely inhibited, preserving the efficiency of ATP synthesis (Slocińska et al., 2016). Among the UCP family, UCP1-UCP3 are the most well-studied. UCP1 is primarily expressed in brown adipose tissue and is responsible for the production of heat through thermogenesis. UCP2 and UCP3 are widely expressed and involved in regulating ROS levels, protecting against oxidative damage and balancing _m_Ca^2+^ uptake (Trenker et al., 2007; Azzu and Brand, 2010). Electrophysiological analysis has proven that UCP2 and UCP3 modulate MCU-dependent Ca^2+^ current in mitoplasts (Bondarenko et al., 2015). On the other hand, UCP4, UCP5, and UCP6 are mainly expressed in the central nervous system and carry out the transport of thiosulfate, sulfate, and inorganic ions (Monteiro et al., 2021).

Apart from their traditional role in proton gradient and ROS homeostasis, UCPs are implicated in several mitochondrial quality control pathways, such as mitophagy and MAD. UCP2 and UCP3 are substrates for ubiquitination and proteasome degradation (Mookerjee and Brand, 2011). Compared to UCP1, UCP2 protein has a very short half-life which is rapidly degraded by the ubiquitin-proteasome system (Azzu and Brand, 2010). The implication of UCP2 degradation in diseases is not yet fully understood. UCPs are also known to be involved in redox signaling and homeostasis. For instance, UCP1 is regulated by ROS levels through sulfonylation of the Cys253 residue, increasing thermogenesis (Oelkrug et al., 2010; Chouchani et al., 2016). UCP2/3 can be modulated by glutathionylation, where GSH is conjugated to cysteine residues. When mitochondrial ROS levels are low, UCP2/3 remains inactive in the presence of glutathionylation. Elevated ROS levels turn on GSH, inducing de-glutathionylation, and subsequent stimulation of UCP2/3 (Mailloux et al., 2011), regulating ROS in a cell. Overall, UCPs play a critical role in regulating energy metabolism, redox homeostasis, mitochondrial quality control pathways, and redox signaling. However, further research is needed to fully understand the implications of UCP degradation in diseases and the broader implications of UCPs in redox signaling and homeostasis.

#### 2.2.10 Other mitochondrial ion transporters

Mitochondria transport various metal ions, including magnesium (Mg^2+^), zinc (Zn^2+^), iron (Fe^2+^), and manganese (Mn^2+^). Mg^2+^ is important for respiratory function, and its concentration is required for Mg^2+^-sensitive matrix enzymes (e.g., pyruvate dehydrogenase) and transporters (e.g., Ca^2+^ uniporter) (Pradhan et al., 2011; Yamanaka et al., 2016). Mitochondrial Mg^2+^ levels are tenfold greater than the cytoplasm, mediating ADP/ATP exchange (Gout et al., 2014) and participating in the release of cytochrome c. The effects of Mg^2+^ on mitochondrial functions emphasize energy metabolism, mitochondrial Ca^2+^ processing, and apoptosis (Pilchova et al., 2017). The transporter of Mg^2+^ of mitochondrial RNA splicing protein 2 (MRS2) channel uptakes Mg^2+^ selectively (Schindl et al., 2007; Yamanaka et al., 2016) and expression levels of MRS2 are linked to the levels of Mg^2+^ (Piskacek et al., 2009). The impaired Mg^2+^ in cells and mitochondria has been studied to be implicated in the neurodegenerative process of Parkinson’s disease (Shindo et al., 2015).

Zn^2+^ is one of the most abundant ions in mitochondria as compared with other subcellular organelles (Sun et al., 2016) and is important for maintaining the antioxidant status of a cell (Adebayo et al., 2016). However, excessive Zn^2+^ within the mitochondria can lead to negative consequences such as loss of MMP, increased ROS, and reduced ATP levels (Pochwat et al., 2015). Studies on MCU knockout mice indicate that Zn^2+^ is transported into mitochondria via MCU (Ji et al., 2020), and it is suggested that the regulatory proteins MICU1 and MICU2 indirectly modulate the transport of both Zn^2+^ and Ca^2+^ levels. To better understand the compartment-specific levels of Zn^2+^ in mitochondria, further investigation is needed.

Mitochondria play a crucial role in iron metabolism, serving as the primary site for hemoglobin production, heme biosynthesis, and Fe-S cluster protein assembly. Mitochondrial solute carrier protein MFRN (SLC25A37) has been proposed to transport iron ions into the MM. Disruptions in MFRN function can lead to severe hypochromic anemia and stagnant red blood cell maturation (Finoshin et al., 2020; Seguin et al., 2020). DMT1, a divalent metal transporter protein located on the mitochondrial outer membrane, has also been shown to transport iron ions (Wolff  et al., 2018). However, little is known about the mitochondrial iron ion efflux channels/transporters and further investigation is needed in this area.

Mitochondria generates ROS which can be detrimental to cells. Therefore, it is essential to remove ROS, and this process relies on SOD (Wang et al., 2018). In mammals, Mn^2+^ uptake is specifically mediated by cellular SLC39A8 (ZIP8) and SLC39A14 (ZIP14) (Park et al., 2015; Tuschl et al., 2016), while SLC30A10 (ZnT10) regulates Mn^2+^ efflux from cells (Mercadante et al., 2019). However, the mechanism of Mn^2+^ transport in mitochondria is still unclear, although it has been proposed that Mn^2+^ uptake from the cytosol to mitochondria may be mediated via MCU or MFRN1 (Liu et al., 2021). Though altered Mn^2+^ homeostasis and toxicity have been reported in neurodegenerative diseases, whether this is associated with mitochondrial Mn^2+^ transport remains to be explored.

#### 2.2.11 Mitochondrial permeability transition pore (MPTP)

The MPTP is a large conductance channel protein located in the IMM. Under physiological conditions, it facilitates the exchange of small metabolites between the matrix and the cytoplasm through its “open and shut” conformation. However, under pathological or stress conditions, the MPTP can transition into a high-conductance state, allowing the uncontrolled access of solutes into the MM, leading to the permeability transition (PT). PT is a reversible process but is tightly controlled and activated by various factors such as Cyclophilin D (CyPD), ROS, matrix Ca^2+^ accumulation, and low ΔΨm (Hodge and Colombini, 1997). The MPTP is a supramolecular non-selective channel that forms at the intersections between the outer and inner mitochondrial membranes. It can transport metabolites up to ∼1,500 Da molecular weight, provided the diameter of the pore is ∼3 nm. The opening of the MPTP can be triggered by Ca^2+^ overload, depolarization of the transmembrane potential, ROS, and Pi (Brustovetsky and Klingenberg, 1996). Inhibitors of the MPTP include Mg^2+^, adenine nucleotides, and mild acidic matrix pH. There are multiple proteins proposed to be the MPTP. Earlier, it was believed that the ANT and FoF1-ATP synthase were responsible for pore formation, but recent evidence has shown that MPTP formation can occur in their absence (Gerle, 2016; Carraro et al., 2018; Karch et al., 2019; Gerle, 2020). SPG7 is identified as a potential MPTP core component in a targeted screening (Shanmughapriya et al., 2015). Therefore, the molecular identify of MPTP is still debated and there is a possibility of multiple proteins acting as MPTP based on upstream signal and cell types. CyPD, a protein found in the MM, can also modulate the opening of the MPTP. PTMs of CyPD have been linked to pore formation, and protein kinases like AKT, cdk5, ERK, PKA, PKC, and PKG can facilitate CyPD interaction with the MPTP through Ser/Thr phosphorylation (Gerle, 2016; Carraro et al., 2018; Karch et al., 2019; Gerle, 2020).

## 3 Pathophysiological implications

Biological cell systems rely on uninterrupted directional flow through metabolic pathways to convert organic molecules into structural entities and energy supply from the mitochondrion. Maintaining electroneutrality within the mitochondrion is essential for regulating the electric field and mitochondrial membrane potential, which is achieved through the prompt exchange of metabolites by carriers or transporters. Mitochondria perform several key cellular activities, including ATP generation (Wang and Youle, 2009), Ca^2+^ and ROS homeostasis (Bernardi, 1999; Murphy, 2009), cell death, and ion transport metabolism (Tzameli, 2012). Dysfunction in these activities has been associated with various pathophysiological conditions, such as cancer, neurodegenerative, cardiovascular, inflammatory, metabolic diseases and others. Numerous studies have demonstrated the implications of mitochondria in cardiovascular (Limongelli et al., 2012), neurodegenerative (Karbowski and Neutzner, 2012), inflammatory (Vringer and Tait, 2019), and metabolic diseases (Joseph et al., 2012). Given the crucial role of mitochondrial transporters and channels in modulation of mitochondrial activities, their dysregulation may contribute to pathophysiological implications observed. Here, we briefly discuss the involvement of mitochondrial channels and transporters in pathophysiology.

Mitochondria have been implicated in cancer progression, exhibiting several cancer hallmarks, including increased cellular proliferation, cell death resistance, induction of angiogenesis, and metabolic reprogramming. The outer mitochondrial anion channel, VDAC, is overexpressed in cancerous cells and is associated with hexokinase 1 (HK1) and hexokinase 2 (HK2) (Wolf et al., 2011; Rosa and César, 2016; Shoshan-Barmatz et al., 2019). VDAC also plays a role in apoptosis, as its interaction with the BAK/BAX complex results an increase in VDAC pore size, leading to the release of cytochrome c and triggering apoptosis (Banerjee and Ghosh, 2004). VDAC opening is also responsible for the release of mtDNA fragments and the onset of lupus-like disease (Kim et al., 2019). Additionally, UCPs, which are major antioxidants in mitochondria, are involved in cancer by favoring oxidative metabolism and AMPK activation in cancerous cells (Robbins and Zhao, 2011). Furthermore, members of the CLIC family, CLIC1 to CLIC6 are also involved in pathological conditions, like cancer initiation and progression (Hernandez-Fernaud et al., 2017), cardiac dysfunction (Takano et al., 2012), and Alzheimer’s Disease (AD) (Milton et al., 2008). For instance, CLIC5 is the first Cl^−^channel discovered in the IMM (Ponnalagu et al., 2016), is expressed in hepatocellular carcinoma and participates in the invasion and migration of tumors (Flores-Téllez et al., 2015). CLIC5 is also overexpressed in acute lymphoblastic leukemia (Neveu et al., 2016). The ubiquitous nature of the CLIC proteins and their involvement in various physiological processes make them a potential target for studying pathological conditions, including cancer. Another ion exchange molecule, LETM1 is also found to be highly expressed in various human malignancies with low survival rates such as lung, ovary, colon, breast, stomach and esophagus (Piao et al., 2019; Wang et al., 2020; Piao et al., 2020). Additionally, reduced NCLX levels are associated with human colorectal tumors, indicating that the regulation of _m_Ca^2+^ can be a novel therapeutic approach in metastatic colorectal cancer (Pathak et al., 2020).

Mitochondrial dysfunction and disrupted Ca^2+^ homeostasis play significant roles in brain and neurodegenerative diseases. Our previous research has demonstrated that impaired _m_Ca^2+^ efflux contributes to _m_Ca^2+^ overload, accelerating the progression of Alzheimer’s disease (AD) and leading to cognitive decline in 3xTg-AD mouse models expressing amyloid beta (Aβ) and tau proteins. Notably, restoring neuronal expression of NCLX in 3xTg-AD mice effectively mitigates cognitive deficits and neuropathology (Jadiya et al., 2019). Moreover, our recent findings reveal that the loss of neuronal NCLX expression induces AD-like dysfunction in aged mice without any genetic predisposition to AD or neurodegenerative conditions (Jadiya et al., 2023). This dysfunction includes memory impairment, accumulation of Aβ plaques, tau hyperphosphorylation, oxidative stress, and synaptic integrity loss (Jadiya et al., 2023). These observations highlight the significant contribution of neuronal NCLX loss and resulting _m_Ca^2+^ overload to neurodegenerative pathology and age-associated cognitive decline. Furthermore, our study demonstrates that specific deletion of MCU, a protein responsible for _m_Ca^2+^ uptake, in 3xTg-AD mice reduces Aβ and tau pathology, synaptic dysfunction, and cognitive decline (Jadiya et al., 2021). Additionally, recent investigations reveal that a loss-of-function mutation in NCLX is associated with severe mental retardation and that the absence of NCLX leads to synaptic dysfunction and deficits in long-term plasticity (Stavsky et al., 2021). In addition, phosphorylation of NCLX by protein kinase A (PKA) has been found to enhance its ability to facilitate the efflux of _m_Ca^2+^, effectively preventing the degeneration of neurons lacking PINK1, a cellular model of Parkinson’s disease (PD) (Kostic et al., 2015). Interestingly, a recent study has unveiled that increased _m_Ca^2+^ uptake, mediated by ERK1/2-dependent upregulation of MCU, contributes to dendritic injury in a model of late-onset familial PD harboring a mutation in Leucine-Rich Repeat Kinase 2 (LRRK2). In various studies, the inhibition of MPTP has shown beneficial effects in mitigating neuronal cell death caused by glutamate excitotoxicity, premature aging, traumatic brain injury, ischemia-reperfusion injury, hepatic IR injury, Parkinson’s Disease, and Alzheimer’s Disease (Uchino et al., 1995; Schinder et al., 1996; Caspersen et al., 2005; Du et al., 2008; Hånell et al., 2015; Morciano et al., 2017; Ludtmann et al., 2018; Panel et al., 2019; Zhou et al., 2019). These findings highlight the potential therapeutic implications of targeting MPTP in neurodegenerative conditions. Furthermore, we have revealed reduced expression of proteins associated with mitochondrial calcium uptake, such as MICU1 and MCUB, in the frontal cortex of both sporadic AD patients and 3xTg-AD mice (Jadiya et al., 2019). Loss of MICU1 due to homozygous deletion of exon 1 has been implicated in sporadic neurological and muscular disorders characterized by _m_Ca^2+^ overload, impaired metabolism, early muscle weakness, myofiber damage, cognitive impairment, and extrapyramidal movement disorders (Logan et al., 2014; Lewis-Smith et al., 2016; Debattisti et al., 2019). MICU1 variants have also been associated with congenital brain malformations featuring white matter abnormalities, cerebellar dysplasia, and acute encephalopathy (Wilton et al., 2020). Additionally, patients carrying nonsense mutations in the *MICU1* gene exhibit myopathy accompanied by extrapyramidal symptoms (Kohlschmidt et al., 2021). Studies in mice with *Micu1* deletion have further demonstrated significant ataxia and muscular defects (Antony et al., 2016; Liu et al., 2016). Additionally, reduced expression levels of UCP2, UCP4, and UCP5 have been observed in patients with AD, which upregulates nitric oxide synthases and compromises mitochondrial functions (de la Monte and Wands, 2006; Finoshin et al.). Moreover, LETM1, a contributing factor in PD and epilepsy, plays a role in calcium handling through constitutive phosphorylation by PINK1 under normal physiological conditions (Huang et al., 2017). In epileptic patients, reduced expression of LETM1 is observed, leading to mitochondrial swelling and increased seizure duration and frequency (Zhang et al., 2014). Collectively, these findings provide compelling evidence linking dysregulation of _m_Ca^2+^ exchange to various neurodegenerative diseases.

Dysregulated Ca^2+^ handling in mitochondria is also implicated in various cardiovascular diseases such as cardiac hypertrophy, cardiomyopathies, arrythmia, myocardial infarction, and cardiac IR injury (Bonora et al., 2019). MCU/mtCU plays a crucial role in the heart’s ability to respond to acute increases in workload and regulates the opening of the mitochondrial permeability transition pore (Kwong et al., 2015; Luongo et al., 2015). Several studies report that all subunits/partners of mtCU complex assist in maintaining physiological Ca^2+^ levels in cells. MCUB, a negative regulator of mtCU complex has been found to protect against cardiac injury by reducing Ca^2+^ influx in mitochondria and inhibiting MPTP opening after IR injury to lessen pathological remodeling (Lambert et al., 2019; Huo et al., 2020). Another important component is MCUR1, which binds to MCU and EMRE to serve as a scaffold protein for the assembly of the MCU complex. We have reported that mouse cardiomyocytes and endothelial cells lacking MCUR1 exhibit severe impairment in _m_Ca^2+^ uptake (Tomar et al., 2016). Collectively, these studies highlight the essential role of the MCU transporter in facilitating cardiac adaptation to acute physiological stress.

Diabetes mellitus is a growing healthcare disease worldwide that affects multiple organs and tissues, leading to multiple pathologies, such as cardiovascular disease, leading to structural and functional abnormalities in the absence of major coronary artery disease, hypertension, a valvular or neuromuscular disease called diabetic cardiomyopathy (Rubler et al., 1972). Studies have shown increased levels of MCUB protein and decreased levels of MCU protein in the hearts of mouse models of both type 1 (Suarez et al., 2018) and type 2 (Bækkerud et al., 2019) diabetes as well as in mouse neonatal cardiomyocytes exposed to hyperglycemia (Diaz-Juarez et al., 2016). Furthermore, we have shown that CRISPR/Cas9 mediated deletion of MCU in mouse liver and in *Danio rerio* (zebrafish) prevents _m_Ca^2+^ uptake, delays cytosolic Ca^2+^ clearance, impairs OXPHOS, and leads to hepatic lipid accumulation through dephosphorylation of AMPK by protein phosphatase-4 (Tomar et al., 2016). These findings highlight the involvement of _m_Ca^2+^ handling in the pathogenesis of diabetes-related complications.

Mitochondrial dysfunction is commonly associated with inflammatory responses and is implicated in the development of various diseases. Mitochondrial transporters play a crucial role in modulating inflammatory responses (Ball et al., 2011). For example, the activation of the NLRP3 inflammasome complex via the VDAC indicates its involvement in the inflammatory response (Zhou et al., 2011). UCP2, another mitochondrial transporter, is involved in regulating the inflammatory response by increasing ROS levels in macrophages (Ball et al., 2011). Downregulation of UCP2 is associated with a pro-inflammatory response in autoimmune encephalomyelitis, where T-cell proliferation and B-cell response is increased in *Ucp2* ^*−/−*^ mice (Vogler et al., 2006). CLICs, when translocated to the cellular membrane, enhance inflammasome assembly, interleukin-1β release, and caspase-1 activation, contributing to inflammation. (Tang et al., 2017). Additionally, elevated levels of myocardial UCP2 under hypoxic conditions can induce ischemic insult (Tian et al., 2018).

Mitochondrial channels and transporters play significant roles in both physiological and pathophysiological conditions. However, due to their dual conductance states (open and closed), establishing a clear structure-function relationship during disease conditions can be challenging. Further studies are necessary to understand the nature of their conductance states in healthy and diseased states. Such research is essential for designing potential therapeutics and advancing medical science.

## 4 Future outlook of mitochondrial transporters or channels: avenues for the development of potential therapeutic target

Mitochondrial transporters and channels play a critical role in maintaining cellular homeostasis, cell death, and signaling. As carriers of metabolites, they help maintain the balance between energy supply and demand, making them crucial determinants of a cell’s survival. Although the functional and biophysical characteristics of these transporters have been studied extensively, their molecular identity and regulatory mechanisms at the molecular level remains largely unknown. Understanding how these channels are regulated could lead to the identification of promising therapeutic targets for mitochondria-related diseases. One major challenge in targeting PTMs associated with channels is that they share biophysical properties with plasma membrane channels. However, a molecular understanding of channels during both physiological and disease states can provide insight into designing new therapeutic approaches for various diseases such as cancer, diabetes, cardiovascular, and neurodegenerative diseases. Research efforts should be directed towards exploring the structural conformations of these channels, their transport mechanisms, and the identification of functional interactors. Such studies could shed light on the molecular mechanisms underlying mitochondrial transporters and channels and pave the way for the development of new and effective treatments for a range of diseases.
